# Single Nucleotide Polymorphisms Associated with Rheumatoid Arthritis in Saudi Patients

**DOI:** 10.3390/jcm12154944

**Published:** 2023-07-27

**Authors:** Maha Daghestani, Nashwa Othman, Mohammed A. Omair, Fahidah Alenzi, Maha A. Omair, Eman Alqurtas, Shireen Amin, Arjumand Warsy

**Affiliations:** 1 Department of Zoology, College of Science, Center for Science and Medical Studies for Girls, King Saud University, Riyadh 11451, Saudi Arabia; mdaghestani@ksu.edu.sa; 2 Central Laboratory, Center for Science and Medical Studies for Girls, King Saud University, Riyadh 11451, Saudi Arabia; nashwao@ksu.edu.sa (N.O.); samin@ksu.edu.sa (S.A.); aswarsy@gmail.com (A.W.); 3 Rheumatology Unit, Department of Medicine, College of Medicine, King Saud University, Riyadh 11451, Saudi Arabia; momair@ksu.edu.sa (M.A.O.); equrtas@gmail.com (E.A.); 4 Department of Clinical Science, College of Medicine, Princess Nourah bint Abdulrahman University, Riyadh 11671, Saudi Arabia; 5 Department of Statistics and Operations Research, College of Sciences, King Saud University, Riyadh 11451, Saudi Arabia; maomair@ksu.edu.sa

**Keywords:** rheumatoid arthritis, single nucleotide polymorphism, Saudi patients, RA genetics

## Abstract

Rheumatoid arthritis (RA) is a complex, multifactorial disorder with an autoimmune etiology. RA is highly heritable and is associated with both human leucocyte antigen (HLA) and non-HLA genes. We investigated the associations of 33 single nucleotide polymorphisms (SNPs) with RA in the Saudi population. Methods: This study included 105 patients with RA and an equal number of age- and sex-matched controls. The patients with RA attended outpatient clinics at King Khalid University Hospital in Riyadh, Saudi Arabia. Blood samples were collected, and DNA was extracted using Qiagen kits. Primers were designed for the 33 selected SNPs using the MassEXTEND primers program, and samples were genotyped on the Sequenom MassARRAY iPLEX platform. The allele frequencies and genotypes were determined for each SNP, and the results obtained for the patients were compared to those for the controls. Results: The allele and genotype frequencies of six SNPs were significantly associated with RA: rs1188934, rs10919563, rs3087243, rs1980422, rs10499194, and rs629326. The minor alleles of rs1188934, rs10919563, rs10499194, and rs629326 were protective, with odds ratios of 0.542, 0.597, 0.589, and 0.625, and *p*-values of 0.002, 0.023, 0.013 and 0.036, respectively. In addition, the heterozygote frequencies of two SNPs (rs6859219 and rs11586238) were significantly higher in the controls than in the patients. Conclusions: There is considerable heterogeneity in the genetics of RA in different populations, and the SNPs that are associated with RA in some populations are not in others. We studied 33 SNPs and only eight were associated with RA. The remaining SNPs showed no allelic or genotypic associations with RA.

## 1. Introduction

Rheumatoid arthritis (RA) is a common autoimmune disorder, affecting about 0.5%–1% of the global population. It is a complex, multifactorial disorder that arises from the activation of several pathological pathways by genetic variants, environmental factors, and immune dysfunctions. In addition, the interaction of various random events has also been proposed [1]. These events cause inflammatory responses that lead to synovitis of both small and large joints and may destroy bone and cartilage. If left untreated, these complications may cause severe pain, swelling, deformity, joint disability, and reduced quality of life [2]. Studies have shown that RA has a genetic component and shows high heritability (50–60%), with genetic variations in the human leukocyte antigen (HLA) genes alone accounting for almost 10–40% of its heritability [1]. Non-HLA genes also play a significant role, and numerous studies have shown the involvement of several genes in RA susceptibility [3]. Over four decades of research have identified many genetic susceptibility loci, with genome-wide association studies (GWAS) indicating susceptibility loci in both coding (exons) and non-coding (introns and untranslated regions) regions of some genes (exons) and in the intergenic regions [4]. It has been over a decade since the first GWAS on RA was reported, and there has been an exponential increase in the number of genes associated with RA development [5,6]. Recent large meta-analyses of African Americans, Europeans, and other populations have identified >100 loci that influence RA susceptibility, and have also expanded knowledge about the factors contributing to or protecting against RA development [7,8,9,10]. GWASs have also shown that only 20% of the risk loci are located in coding sequences, with the rest occurring in non-coding regions [7]. In addition, epigenetic studies have indicated the contribution of many causal genetic components. However, extensive studies are needed to identify the missing link between genetic risk factors and causal genetic components [11]. A comprehensive database has been developed for RA and related disorders that contains known RA-related polymorphisms (RADB) [12]. It was prepared from 686 published reports from 68 countries and contains 3235 polymorphisms associated with 636 genes (available at: http://www.bioapp.org/RADB (accessed on 1 December 2022)). Like many other studies, it reported that the strongest genetic association for RA is with major histocompatibility complex, class II, DR beta 1 (HLA-DRB1) alleles on chromosome 6p21. The list of single nucleotide polymorphisms (SNPs) associated with RA is long and includes numerous non-HLA genes, including *TRAF1*, *STAT4*, *CTLA4*, *IRF5*, *CCR6*, *PTPN22*, *IL23R*, *PADI4*, *GPC5*, *FCRL*, *RBFOX1*, *IL6R*, *SPRED2*, *PXK*, *RBP1*, *TAGAP*, *TAGAP*, *CCL3*, *CARD8*, *CD40*, *IRAK1*, *GATSL3*, and *MTHFR* [1,3,10,13,14]. Furthermore, it has also become apparent from several meta-analyses that different populations have different susceptibility loci which, along with environmental factors, contribute to RA development. Some variants are common and occur at a higher frequency in several populations, while others are rare [14,15,16]. Laufer et al. [9] identified novel susceptibility genes in African-American populations that were not found to be associated with RA in Europeans, East Asians, or the Japanese [10]. Some polymorphisms identified in genic and nongenic regions increase susceptibility to RA, while others are protective and prevent or delay RA development [13]. Some functional studies have shown that depending on their location, genetic variants within or outside genes may affect the stability or translation rate of synthesized mRNA or the post-translational modification, structure, activity, or stability of the synthesized protein [10]. Some polymorphisms influence transcription factor binding to the promoter to initiate or inhibit gene expression. Examples of gain-of-function and loss-of-function of the associated genes have been reported [10]. Another interesting finding is that these polymorphisms are associated with clinical RA features. The variants may differ in their associated RA susceptibility, age of onset, sex bias, serum factors, or synovial fluid factors (e.g., rs1800896 [–1082G/A], PTPN22 rs2476601, IL8 rs2227306 [781C/T], IL6 rs1800795 [–174G/C], IL2 [–330G/T], and TNFA rs1800629 [–308A/G]) [17,18,19]. Interestingly, many variants that are positively or negatively associated with RA development also affect the development of other autoimmune disorders. This study investigated 33 SNPs in Saudi patients with RA and healthy controls to identify the SNPs associated with RA in Saudi Arabia. Our findings show that six of these SNPs have a strong allelic association with RA, while two other SNPs also have specific genotypic associations with RA.

## 2. Materials and Methods

Patients and controls: This study was designed as a cross-sectional study, and was conducted at the Research Center at The Center for Female Medical and Science Students, King Saud University, Riyadh, Saudi Arabia. This study included patients with RA who attended the outpatient clinics of the co-investigator (MO) at KKUH. The purpose of this research was explained to them, and only those who volunteered and signed informed consents were included in this study. The sampling criterion required the inclusion of every consecutive patient with RA, diagnosed and classified according to the 2010 American College of Rheumatology/European League Against Rheumatism classification criteria [20]. The criteria overlapped with other connective tissue diseases, including end-stage organ disease or malignancy. The controls were age- and sex-matched healthy individuals from the same general population, with no signs or family history of RA in first-degree members or other major health problems. The demographic details, disease duration, seropositivity, and information about the current therapies for each patient were recorded from the medical records. Disease activity was calculated using swollen joint counts, tender joint counts, and the erythrocyte sedimentation rate (ESR). Disease state was assessed using the Disease Activity Score at 28 joints-ESR (DAS-28) as follows: remission, DAS-28≤2.6; low disease activity, 2.6<DAS-28≤3.2; moderate disease activity, 3.2<DAS-28≤5.1; and high disease activity, DAS-28>5.1 [21]. Sample collection and preparation: The study population comprised only Saudis and included 210 individuals (patients with RA = 105; controls = 105). A blood sample (3 mL) was collected from each patient and healthy control with venipuncture into EDTA tubes and was used for DNA extraction with a Qiagen kit. The DNA concentration was determined using a Nanodrop spectrophotometer (Thermo Fisher Scientific, Wilmington, DE, USA). Genomic DNA extraction: DNA was extracted from the blood samples using Qiagen kits according to the manufacturer’s recommended protocol. The concentration of the extracted DNA from each individual was determined by measuring the optical density at 260 nm (OD_260_) using a NanoDrop ND-1000 spectrophotometer (NanoDrop 2000, Thermo Fisher Scientific). The final DNA concentration in each sample was standardized to 15–20 ng/μL by dilution. An OD_260_/OD_280_ ratio was obtained using the NanoDrop, and a ratio close to 1.8 indicated a relatively pure DNA sample. Design of primers and SNP selection: A literature search was carried out using PubMed for “genetics of rheumatoid arthritis in different ethnic groups”. Thirty-three SNPs in different genes were randomly selected for inclusion in this study. The chromosome/position, associated gene (if any), mutation, minor allele, and location of the 33 SNPs investigated during this study are presented in Table 1 (all the selected SNPs were located in or around a gene except for six). The NCBI dbSNP website (https://www.ncbi.nlm.nih.gov/snp (accessed on 1 December 2022)) was used to confirm each SNP. PCR primers were designed for each SNP using the MassEXTEND primers program. The primers used to amplify the 33 SNPs were synthesized using Aracure (Wilmington, DE, USA). SNP genotyping: The software and equipment required for SNP genotyping were purchased from Sequenom. Genotyping was performed using the Sequenom MassARRAY iPLEX platform and the iPLEX Gold SNP Genotyping Kit using the manufacturer’s recommended conditions. The forward and reverse primers used for the PCR and the extended primer used for the Mass Array studies of the 33 SNPs during this investigation are presented in Table 2. The PCR master mix contained 0.1 M of the forward and reverse PCR primers, 2 mM of magnesium chloride, 0.5 U of the HotStarTaq enzyme, 500 M of deoxynucleoside triphosphates, and genomic DNA. An initial denaturation was conducted at 95 °C for 2 min, followed by 45 cycles of denaturation at 95 °C for 30 s, annealing at 56 °C for 30 s, and extension at 72 °C for 1 min. These 45 cycles were followed by a final extension at 72 °C for 5 min. Using mass-modified dideoxynucleotides, extension primers were attached directly next to the target SNP sites and extended by one nucleotide base onto the SNP site in the iPLEX reaction, which used the final PCR products. The assay procedure included a first locus-specific PCR reaction, then a single base extension using the dideoxynucleotide terminators of an oligonucleotide primer that annealed directly upstream of the desired polymorphism site. Utilizing SpectroCLEAN resin, the final products were desalted. Using an RS1000 Nanodispenser, the cleaned extension products were applied to 96 SpectroCHIP arrays. The array was then put into a MassARRAY Compact 96 mass spectrometer, and the software SpectroAcquire was used to acquire the generated spectra. Automated allele calling and data analysis were performed using the MassARRAY Typer 4.0 software (version 4.0, Sequenom). The alleles were differentiated based on differences in their masses on the matrix-assisted laser desorption/ionization-time of flight mass spectrometer. All analyses were performed in duplicate, and negative controls and blanks were included for accuracy. After genotyping on the MassARRAY, a genotype was assigned for each SNP in each individual, and a Microsoft Excel (Seattle, WA, USA) flowsheet was used to enter the data. **Statistical analysis:** The genotypes were recorded for each SNP in an Excel spreadsheet, and the genotype and allele frequencies were calculated for the patients and control groups. The odds ratios (ORs), 95% confidence intervals (CIs), chi-square (χ^2^) tests, and *p*-values were calculated using the link (http://ihg.gsf.de/cgi-bin/hw/hwa1.pl (accessed on 1 December 2022)), and the results were compared between the patient and control groups. A *p*-value < 0.05 was considered statistically significant. All the analyses were conducted using IBM SPSS software (version 22).

## 3. Results

This study included adult patients with RA (mean age = 47 ± 14.49 years) and an equal number of age- and sex-matched controls. Their demographic data and essential clinical features are provided in Table 3. The patients’ average RA duration was 7.4 ± 5.26 years, and 80.8% were seropositive. The allele frequencies and genotypes were determined and compared between the patient and control groups. The minor allele frequencies were calculated for the patient and control groups, and the significance of differences between them was determined. Table 4 presents the frequency of the minor alleles (MAF) of the 33 studied SNPs in the RA patients and controls. It includes the obtained odds ratio (OR), 95% confidence interval (CI), chi-square (χ^2^), and *p*-value between the results of the two studied groups. Six SNPs were significantly associated with RA: rs1188934, rs10919563, rs3087243, rs1980422, rs10499194, and rs629326. The minor alleles of rs1188934, rs10919563, rs10499194, and rs629326 were protective, with ORs of 0.542, 0.597, 0.589, and 0.625, and *p*-values of 0.002, 0.023, 0.013, and 0.036, respectively. Table 5 presents the genotype and allele frequencies of the six significantly associated SNPs in the patient and control groups. It also includes two SNPs (rs6859219 and rs11586238) with significantly higher heterozygote frequencies in the control group compared to the patient group; their homozygote and allele frequencies did not differ significantly.

## 4. Discussion

RA is a complex multifactorial disorder. In addition to environmental and autoimmune factors, genetic variants play a significant role in its development. Numerous studies have investigated the genetic basis of RA and found heterogeneity in the associated genes and SNPs in different populations. Genetic factors are reported to account for up to 50–60% of RA susceptibility [22]. Due to the polygenic nature of multifactorial disorders, several genes, each with a small effect which may be additive, may lead to disease development in the presence of predisposing environmental factors. Heterogeneity is also reported in the genetic basis of RA in different populations, and polymorphisms at some loci are more frequently encountered in diverse populations than others. This study genotyped 33 SNPs in Saudi patients with RA to investigate their association with RA. The aim was to identify one or more SNPs that could be used as predictors of RA susceptibility in the Saudi population. Table 5 shows that the genotypes and alleles of six of the investigated SNPs (rs1188934, rs10919563, rs3087243, rs1980422, rs6859219, and rs10499194) were significantly associated with RA in Saudis. In addition, the genotypes, but not alleles, of two other SNPs (rs629326 and rs11586238) were significantly associated with RA. Interestingly, these SNPs have also been reported to have an association with RA in different populations. The remaining 25 SNPs did not show any significant differences in genotype or allele frequencies between the patient and control groups despite having been shown to be associated with RA susceptibility or resistance in several studies. The SNP rs10919563 (G>A) was significantly associated with RA in Saudi patients. Both its allele and genotype frequencies differed significantly between the patient and control groups. The wildtype G allele was present at a significantly higher frequency in the patient group than in the control group (*p* = 0.023). The homozygous GG genotype had an OR of 3.232, a χ^2^ of 7.24, and a *p*-value of 0.007. The mutant A allele was significantly protective (OR = 0.597, 95% CI = 0.383–0.932, χ^2^ = 7.24, *p* = 0.007). The SNP rs10919563 involves a G>A transition and is located in an intron in the protein tyrosine phosphatase receptor type C (*PTPRC*/*CD45*) gene on chromosome 1q31. It encodes a member of the protein tyrosine phosphatase family and plays a role as a signaling molecule, regulating many cellular processes, including differentiation, cellular growth, mitosis, and oncogenic transformation [23]. It was also shown to regulate B- and T-cell antigen receptors and to be involved in cytokine receptor signaling. The frequency of the mutant allele was 0.3196 in the healthy Saudi population. Its frequency in healthy populations worldwide ranges from 0.0758 in the Finnish to 0.5202 in the Esam population in Nigeria, as reported in the NCBI database based on the results of the 1000 Genomes Project (https://www.ncbi.nlm.nih.gov/variation/tools/1000genomes/; accessed on 4 April 2021). Several studies, including a meta-analysis and GWAS, have reported associations between rs10919563 and several autoimmune disorders, including RA [6,24,25,26]. It was significantly associated with anti-cyclic citrullinated peptide antibody-negative RA [27] and juvenile idiopathic arthritis [26]. Its association with RA susceptibility was shown to be irrespective of serological status. Differences among populations have been reported, with an association found in Caucasians, Europeans, and African Americans, but not in West/Central Africans [25,26,27]. The findings of this study show that it is also associated with RA in Saudi Arabians. It would be interesting to compare the prevalence of RA in different populations and to correlate it with the frequency of the minor allele A of rs10919563 in these populations. Studies have also shown a significant association between the susceptibility allele of rs10919563 and the response to anti-tumor necrosis factor (TNF) therapy in patients with RA [28], where the susceptibility allele predicted an improved treatment response to TNF blockade [29]. A strong association with RA was found for rs3087243 (G>A), where the wildtype allele G was highly predisposing to RA (OR = 1.733, 95% CI = 1.235–2.623, χ^2^ = 9.41, *p* = 0.002), and the mutant A allele and its homozygous genotype AA were highly protective (OR = 0.556, χ^2^ = 9.41, *p* = 0.002). This SNP is located downstream of the cytotoxic T lymphocyte-associated protein-4 (*CTLA4*/*CD152*) gene on chromosome 2. CTLA4 is a negative regulator of T-cell proliferation. Studies have reported its association with RA susceptibility in some populations [22,30,31,32,33,34,35] but not others [36,37,38]. A study on individuals of African ancestry found that the markers associated with RA in Europeans were not replicated in African Americans [36]. Interestingly, rs3087243 was shown to be associated with several other autoimmune disorders, including Graves’ disease, diabetes mellitus type 1, autoimmune thyroid disease, ankylosing spondylitis, myasthenia gravis, ulcerative colitis, non-glandular autoimmunity, immune thrombocytopenia, allergic rhinitis, Crohn’s disease, and vitiligo. It also plays a role in the etiopathogenesis of breast cancer and decreases the risk of cervical cancer. rs1980422 (T>C) is a transition mutation near the *CD28* gene on chromosome 2. CD28 plays a role in stimulating T cells as a stimulatory signal transducer and is a common determinant for seropositivity in RA [24]. In an Egyptian study, rs1980422 in CD28 showed a strong association with the entire RA cohort and its seropositive subset [39]. A 2009 meta-analysis of GWASs validated the association of rs1980422 with RA susceptibility [40]. However, other studies (e.g., the study of Luterek-Puszyńska et al. on the Polish population [32]) have shown no association with RA, possibly due to population differences. It is also not significantly associated with the response to anti-TNF biologics in patients with RA [41]. rs1980422 has also been associated with other autoimmune disorders, including primary immune thrombocytopenia. It was confirmed through a meta-analysis that it is associated with juvenile idiopathic arthritis [42], but not with lupus [43]. In the 1000 Genome Project, the frequency of the C allele in normal healthy populations was reported to be the highest (0.3037) in the Toscani population in Italy and lowest in the Telugu population in India (NCBI). In this study, the C allele frequency was significantly higher in Saudi patients with RA (0.5966) than in healthy Saudis (0.3043; OR = 1.545, 95% CI = 1.029–2.322, χ^2^ = 4.41, *p* = 0.013). However, more detailed studies are required to confirm this association. We found rs10499194 in the TNF-alpha induced-protein 3 (*TNFAIP3*) gene on chromosome 6q23.3 to be a polymorphic marker for RA in Saudi patients. A few studies have reported it as a factor associated with decreased RA risk [44]. Several contradictory reports exist in the literature; some show a significant protective influence of the variant allele of rs10499194 [45,46,47,48,49] while others do not [4,47,50]. The *TNFAIP3* gene is induced by TNF and encodes a zinc finger protein that acts as a ubiquitin-editing enzyme. It has two activities, one as a ubiquitin ligase and the other as a deubiquitinase. It is involved in cytokine-mediated immune and inflammatory responses and inhibits nuclear factor-kappa B activation and TNF-mediated apoptosis [51]. rs10499194 involves a C>T transition and is located in an intergenic region approximately 150 kb upstream of *TNFAIP3* and *OLIG3*. It was reproducibly associated with RA in the genome-wide association and case–control samples. This SNP was present in every population included in the 1000 Genome Project. The frequency of its mutant T allele varied from 0.0194 in the Han Chinese in China to 0.3641 in the Gujaratis in India (https://www.ncbi.nlm.nih.gov/variation/tools/1000genomes/ (accessed on 4 April 2021)). Several studies have found its minor allele to be protective against RA [48,52,53]. This variant has also been associated with childhood-onset RA (rheumatoid factor or anti-citrullinated protein antibody-positive juvenile idiopathic arthritis) [54,55]. TNF-α levels are elevated in individuals with RA, and the inhibition of TNF-α is a potent treatment for severe RA [56]. While rs10499194 has been associated with RA, its association is significantly stronger when combined with other SNPs, such as rs6920220, rs13207033, or rs2230926 [53,55,57,58]. The co-existence of rs10499194 and rs13207033 polymorphisms decreased RA risk among Caucasian populations, but not among Asian populations [45]. Interestingly, rs10499194 has been associated with several autoimmune disorders, including type 1 autoimmune hepatitis [58], ankylosing spondylitis [59], primary immune thrombocytopenia [60], Bechet’s disease [61], and systemic lupus erythematosus [47]. Our results for Saudis showed that SNP rs629326 (G>T) is associated with RA, where the mutant T allele was highly predisposing to RA (OR = 1.60, 95% CI = 1.030–2.485, χ^2^ = 4.4, *p* = 0.036). The heterozygous GT genotype was significantly more common in the controls, and the homozygous TT was more common in the RA patients, although the *p*-value did not reach significance. SNP rs629326 is located in an intron in the T-cell activation Rho GTPase-activating protein (*TAGAP*) gene on chromosome 6q25. It has been reported as a susceptibility locus for RA in different populations [62]. The *TAGAP* gene encodes a member of the Rho GTPase-activator protein superfamily and is a Rho GTPase-activating protein. Several GWAS and metagenome SNP analyses have reported that TAGAP is associated with the pathogenesis of several autoimmune diseases, including RA, psoriasis, celiac disease, Crohn’s disease, and multiple sclerosis. TAGAP was shown to be a novel factor required for T helper 17 (Th17) cell differentiation [63]. Th17 cells contribute to TAGAP-associated autoimmune diseases since *TAGAP* is expressed exclusively in activated T cells [64]. SNP rs629326 is 23.61 kb upstream of *TAGAP*, and is involved in T-cell activation. Only a few published studies are related to this SNP. A 2012 article reported a high-density genetic mapping on RA in patients of European ancestry that identified rs629329 as a new susceptibility locus [65]. In 2016, Jiang et al. [66] investigated genetic risk alleles for RA in a Swedish cohort. A study by Tamehiro et al. [63] indicated that TAGAP was a novel factor required for Th17 cell differentiation and suggested that TAGAP potentially represents a novel target for autoimmune disease therapies. The mechanism behind the action of rs629326 is unclear. However, since this SNP is not in an exon, it will not affect the amino acid sequence of the protein product. It might influence gene expression and, hence, the amount of the gene product produced in its presence and absence. It can be confidently stated that increased *TAGAP* expression is a feature of inflammatory diseases, including RA. Therefore, any increase in its expression will also increase susceptibility to such disorders [67]. Many studies have implicated the *STAT4* gene in RA development [68], and several SNPs have been identified [69,70,71]. SNP rs1188934 (A>G) has been investigated in only a few reported studies. It is an intronic variant in the *STAT4* gene on chromosome 2q33, which encodes a member of the STAT family of transcription factors. These factors are phosphorylated by the receptor-associated kinases in response to cytokines and growth factors, forming homo- or hetero-dimers that act as transcription activators. Mutations in and around the *STAT4* gene may affect the binding of growth factors and influence the transcription rate [71]. There are reports that mutations in this gene may be associated with RA and other autoimmune diseases, such as systemic lupus erythematosus. A few studies have suggested that rs11893432 (C/G) in the *STAT4* gene might also predispose individuals to RA [68,70,71]. Our results on the Saudi population confirm this association, since rs1188934 showed significantly different genotype and allele frequencies in patients with RA than in the healthy controls (*p* = 0.002), where the mutant G allele was highly protective, with ORs of 0.542 for the mutant G allele and 1.846 for the wildtype A allele. The GG genotype had an OR of 0.035, a 95% CI of 0.153–0.801, a χ^2^ of 6.42, and a *p*-value of 0.011. The minor G allele has been reported at significantly high frequencies in all populations. The reported frequencies were lowest in the Gujarati Indians (0.2913) and highest in the Yoruban Nigerians (0.6157). The frequency was found to be 0.429 in healthy Saudis. However, the frequency was significantly lower in the patients with RA (0.289; *p* = 0.002). Two SNPs, rs6859219 and rs11586238, showed significant differences in the frequency of their heterozygous genotypes between the patients with RA and the healthy controls. However, their allele frequencies did not differ between the two groups. SNP rs6859219 (C>A) is an intronic transversion mutation in the Ankyrin repeat domain 55 (*ANKRD55*) gene on chromosome 5q11.2. Several intronic SNPs in this protein-coding gene have been associated with autoimmune disorders, including RA, multiple sclerosis, and oligoarticular juvenile idiopathic [6,72]. The ankyrin repeat domains are one of the most common protein–protein interaction domains and are believed to function in complex disorders. In this Saudi study, the mutant A allele was present at a lower frequency in the patients with RA than in the healthy controls, but the difference was not significant. Nevertheless, the heterozygous CA genotype was present at a significantly lower frequency in the patients with RA (0.225; OR = 0.519, 95% CI = 0.287–0.939, χ^2^ = 4.75, *p* = 0.029). Further studies with larger sample sizes may clarify the association of this SNP. Finally, rs11586238 (C>G) is in the *CD2* gene, which encodes a surface antigen expressed on all peripheral blood T cells. This surface antigen interacts with the LFA3 receptor (also known as CD58) on antigen-presenting cells (APC) and helps recognize antigens. In the 1000 Genomes Project, the G allele frequency ranges from 0.0143 in Southern Han Chinese and 0.0365 in the African Caribbeans of Barbados to 0.3297 in the British population of England and Scotland (https://www.ncbi.nlm.nih.gov/variation/tools/1000genomes/; accessed on 4 April 2021). A few studies have reported an association between rs11586238 and RA [27].

## 5. Conclusions

There is considerable heterogeneity in the genetics of RA in different populations. Several polymorphic sites are associated with RA in some populations but not in others. Though we studied 33 SNPs, only eight were associated with RA in Saudis; the remaining 25 showed no genotypic or allelic associations with RA in Saudis, despite the reports of their association with RA in other populations in previous studies. This study has provided valuable data for RA treatment in Saudis and has shown that treatment protocols could be designed using the SNPs showing strong associations with RA.

## Figures and Tables

**Table 1:** The chromosome/position, associated gene (if any), mutation, minor allele, and location of the 33 SNPs investigated during this study.

	SNP	Chromosome/Position	Gene	Mutation	Minor Allele	Location
**1**	rs11586238	1:116720516	None	[C>G]	G	-
**2**	rs1188934	1:90839539	LINC02609	[A>G] A>C	G	ntron_variant, genic_upstream transcript_variant
**3**	rs2240340	1:17336144	PADI4	C>T	T	(intron variant)
**4**	rs2476601	1:113834946	PTPN22 AP4B1-AS1;	[A>T] G	A	Missence variant Intron variant
**5**	rs10919563	1:198731313	PTPRC	[A>G]	A	Intron variant
**6**	rs3766379	1:160837925	CD244	[C/T]	T	Intron variant
**7**	rs3890745	1:2622185	MMEL1	[A>G] T/C	C	Intron variant
**8**	rs13031237	2:60908994	REL	[G>T]	T	Intron variant
**9**	rs3087243	2:203874196	CTLA4	[A/G] G>A	A	500B Downstream variant
**10**	rs1980422	2:203745673	None	C>A/C>T	C	
**11**	rs7574865	2:191099907	STAT4	[T>A/T>G]	T	Intron variant
**12**	rs13315591	3:58571114	FAM107A LOC107984079	T>C	C	Intron variant Non-coding transcript variant
**13**	rs874040	4:26106575	None	G>C	C	None
**14**	rs6859219	5:56142753	ANKRD55	[A/C] C>A	A	Intron variant
**15**	rs10499194	6:137681500	None	C>T	T	None
**16**	rs3093023	6:167120802	CCR6; LOC105378122	[G>A] G>T, G>C	A	Intronic variant Non_coding transcript variant
**17**	rs394581	6:159061489	LOC105378083 v: LOC1122679683	[C/T]	C	Intronic variant prime_UTR variant
**18**	rs5029937	6:137874014	TNFAIP3	[G/T]	T	Intronic variant
**19**	rs548234	6:106120159	None	[C/T]	C	
**20**	rs629326	6:159075681	LOC112267968	[G/T]	G	Intronic variant
**21**	rs10488631	7:128954129	TNPO3	T>C	C	Downstream transcript variant
**22**	rs10739580	9:120933004	None	C>T	C	-
**23**	rs2812378	9:34710263	CCL21	G>A, T>G C>T	G	Upstream variant
**24**	rs3761847	9:120927961	TRAF1	[A/G] G G>A,C	G	Intron variant
**25**	rs4750316	10:6351298	LINC02656	[C>G]	C	Non_coding_transcript_variant
**26**	rs706778	10:6056986	IL2RA	[A/G] C>T	T	Intron variant
**27**	rs2104286	10:6057082	IL2RA	A/G T/C	C	Intron variant
**28**	rs540386	11:36503743	TRAF6	[C>G] C>/T	T	Intron variants
**29**	rs10683701	12:57698305	OS9	[-/ACTT] DEL C>CACTT	-/	Intron variant
**30**	rs1678542	12:57574932	KIF5A	[C/G]	G	Intron variant
**31**	rs763361	18:69864406	CD226	T>[A/C/G] A/T A	C	Missence variant (coding variant)
**32**	rs4810485	20:46119308	CD40	T>A/G G/T---T	T	Intron variant
**33**	rs3218253	22:37148770	IL2RB	[C/T] G>A	A	Intron variant

**Table 2:** Forward and reverse primers used for the PCR and the extended primer for the Mass Array studies used for the investigation of 33 SNPs during this investigation.

	SNP ID	Forward Primer Sequence	Reverse Primer Sequence	Extended Primer Sequence
**1**	rs11586238	ACGTTGGATGGCCTGCTTGAACCTCTTTTG	ACGTTGGATGGGCCCAGATCATGAACAGAC	CCATTGCCTTCAGCATA
**2**	rs1188934	ACGTTGGATGAAACAGATGGCTCAGCAAAC	ACGTTGGATGATCAGGCCCTGACCACTATC	atgaCAAGATTCCTTTTTCTCAGA
**3**	rs2240340	ACGTTGGATGGGACCCTCACCAACCTCTC	ACGTTGGATGTGGTTGGCTTCACTTTGCCG	ACCAACCTCTCCTCTTAC
**4**	rs2476601	ACGTTGGATGACTGAACTGTACTCACCAGC	ACGTTGGATGAGATGATGAAATCCCCCCTC	CCCTCCACTTCCTGTA
**5**	rs10919563	ACGTTGGATGGCATGTTTACAGTATTTCAC	ACGTTGGATGATCCCAGACCAAACATCACC	TTATAGTAATTGCTATAAAATGCATATA
**6**	rs3766379	ACGTTGGATGTTGGATGACAGGCAGAGTTG	ACGTTGGATGACTAGAGAGAGTAACCAGCC	ttagAAGCCCACCAGCCTGAGT
**7**	rs3890745	ACGTTGGATGCCACCTGAGCATTTTGTGAC	ACGTTGGATGTCACCTGGGGAAATTGTTAC	ccttGGGAAATTGTTACAAATCCAGAC
**8**	rs13031237	ACGTTGGATGAAAGCCTTTCCTTACAACTG	ACGTTGGATGGCTTCGAAAACTCTGACTGC	TTTGAAAAAATGGCTCATGT
**9**	rs3087243	ACGTTGGATGTTTCTTCACCACTATTTGGG	ACGTTGGATGCCTGTGTTAAACAGCATGCC	aTTCACCACTATTTGGGATATAAC
**10**	rs1980422	ACGTTGGATGATATCCGCAAGCTATTTTGG	ACGTTGGATGGTCCTCAATTTTCCCAAGTC	aCAAGTCTTTTCATAATACCTGTTTC
**11**	rs7574865	ACGTTGGATGGAGTGTGTATGCAGTAAAAG	ACGTTGGATGAATCCCCTGAAATTCCACTG	gTCCACTGAAATAAGATAACCACTATT
**12**	rs13315591	ACGTTGGATGTGGATGAACAGGGATGTGTG	ACGTTGGATGATCACCTTGCAACGTGCACC	ttctCTGAAAGTGGCAAACAGCTTA
**13**	rs874040	ACGTTGGATGTCCTGATTGTGGCTCGGATG	ACGTTGGATGCCACAGAATCTCCCATAAAC	TGCAAAAGCTGCGTG
**14**	rs6859219_	ACGTTGGATGTACAGTGGTGACCCCTGAC	ACGTTGGATGGTATCTAATCACCTGCCCTG	TCGCTGCCAGTCTCT
**15**	rs10499194	ACGTTGGATGAGCTATCAGTTTCATTACC	ACGTTGGATGCAGACCACACAGTTTCTAGG	aaagGACTACTTTTTGAACAAAAGGGTT
**16**	rs3093023	ACGTTGGATGTTCCTCGCCTTTTATGCACC	ACGTTGGATGGATCCTCTTAGATCTCACTC	tccCTTCCTCAAATTTAAAATCACA
**17**	rs394581	ACGTTGGATGTCCAGCCAGATTTCAGGCTC	ACGTTGGATGAGTCAGAGAGTTCGCCGTAG	ggggCGGCCAAGCAGATAGATAA
**18**	rs5029937	ACGTTGGATGCTTGCCAAAGGAGATTAAGG	ACGTTGGATGACTCACAATTCAATGGGCTG	CCCAAAATATTTATCGTTTGGGG
**19**	rs548234	ACGTTGGATGGGAAATTAGCTGGGCTCTTC	ACGTTGGATGCTCAATCTCTTGCGCTCTTC	ccGCAATTTTTGTCTTCTCTCAC
**20**	rs629326	ACGTTGGATGTTTGTTTCTGACCCACAGCG	ACGTTGGATGACAGAGCAGGACTCCCATCA	ggggAAAGGAACTGCTGTTCT
**21**	rs10488631	ACGTTGGATGGTCTATCAGGTACCAAAGGC	ACGTTGGATGATTCACTGCCTTGTAGCTCG	ctTAGCTCGGAAATGGTTC
**22**	rs10739580	ACGTTGGATGGTGCCTGTTTACAGGTTTT	ACGTTGGATGGATACAGCTTTACTTTCATGG	CTACCACAGAATTATGAATACA
**23**	rs2812378	ACGTTGGATGAAAGCTGGATTTGCTGGCAC	ACGTTGGATGCAGGCCCAGACATATTCAAC	GCAGCTGAGGACTGTCCA
**24**	rs3761847_	ACGTTGGATGATCTGTGGGTCCCTTCTCTC	ACGTTGGATGTTGATGTCCGTGGGAATGAG	tagcGGGTGGTATTGAGGC
**25**	rs4750316	ACGTTGGATGTACGGAAGAGCTGATAAGGG	ACGTTGGATGCCCTCATTGTCACCTAATGG	CACCTAATGGTGGTACT
**26**	rs706778	ACGTTGGATGAGGAGCACAGTGGACCACCT	ACGTTGGATGCCCTGAGGGACTGGTAAATT	gggagGGGACTGGTAAATTTCCATCA
**27**	rs2104286	ACGTTGGATGCCATGCTCAGTAGATCTTAC	ACGTTGGATGGTCATAAGTTGGTGAGGAGG	agcTATAGTCATGGTAACACAAGTC
**28**	rs540386	ACGTTGGATGAGCAGAACTAGTCACTACAG	ACGTTGGATGCCCTAGTGTAGCATAACAGC	GGGCCCTATACCGTATTTTAC
**29**	rs10683701	ACGTTGGATGCCACACACGTATATAATCCG	ACGTTGGATGTCTTGGCCTACTGAAGATAC	AACGAATAACTAGAATACAATGAAGT
**30**	rs1678542	ACGTTGGATGGCAGGCGGAGGAATTTAATG	ACGTTGGATGCATACGCAGGGACTCAAATG	ACCTTTAGCAGCTCTCTATCA
**31**	rs763361	ACGTTGGATGGAGAAGGTTGGATAGTTGAC	ACGTTGGATGGTTTGTCTTTCTAGGCACCC	TAGAAGTCCCATCTCTACC
**32**	rs4810485	ACGTTGGATGAAGTACCTGGCTCCTTCATC	ACGTTGGATGATACCATGGGTCATTCCTGC	GAGGGCTGTAGATTCC
**33**	rs3218253	ACGTTGGATGTGAGGAGACTAAGAAACGGG	ACGTTGGATGAACTGCACCTGACCAGGTTC	ttttcCAACCTCTCACCCAG

**Table 3:** Demographic and clinical characteristics of RA Patients.

Demographic and Clinical Characteristics of RA Patients		Mean ± SD or (%)
**Males: Females *n* (%)**		14.1: 85.9
**Age (±SD) (years)**		47 ± 14.49
**Mean disease duration (±SD) (Years)**		7.4 ± 5.26
**Seropositive *n* (%)**		80.8
**Mean ESR (±SD)**		44.48 ± 30.87
**Mean DAS-28 (±SD)**		4.34 ± 1.29
**Medications**	Prednisolone *n* (%)	2.6
	csDMARDs *n* (%)	30.8
	Biologics± csDMARDs *n* (%)	60.3
	Drug-free remission *n* (%)	7.7

**Table 4:** The frequency of the minor alleles (MAF) of the 33 studied SNPs in the RA patients and controls, and the odds ratio (OR), 95% confidence interval (CI), chi-square (χ^2^), and *p*-value between the results of the two studied groups.

No.	SNP	Minor Allele	MAF Control	MAF RA	OR	95% CI	χ^2^	*p*-Value
**1**	rs11586238	G	49 (22.48)	42 (18.1)	0.762	0.481–1.209	1.33	0.248
**2**	rs1188934	[C/G/T] A/G---G	91 (42.92)	66 (28.95)	0.542	0.365–0.804	9.35	002
**3**	rs2240340	[A/G] C/T---T	105 (48.61)	114 (49.56)	1.039	0.717–1.506	0.04	0.840 (P)
**4**	rs2476601	[A/G] G/A----A	6 (2.75)	8 (3.23)	1.178	0.402–3.449	0.09	0.765 (P)
**5**	rs10919563	[A/G]---A	62 (31.96)	46 (21.9)	0.597	0.383–0.932	5.2	0.023
**6**	rs3766379	[C/T] T	80 (38.46)	87 (37.83)	0.973	0.662–1.432	0.02	0.891 (P)
**7**	rs3890745	[A/G] T/C---C	70 (33.02)	67 (28.88)	0.824	0.550–1.233	0.89	0.346 (P)
**8**	rs13031237	[G/T]—T	37 (17.96)	49 (20.76)	1.197	0.744–1.924	0.55	0.458
**9**	rs3087243	[G/A] G	87 (41.04)	129 (55.6)	1.799	1.235–2.623	9.41	*p* = 0.0021 (P)
**10**	rs1980422	[A/C/T] T/C---C	56 (30.43)	142 (59.6)	1.545	1.03–2.32	4.41	0.036
**11**	rs7574865	[G/T] T	60 (30)	58 (24.37)	0.752	0.492–1.148	1.75	0.186 (P)
**12**	rs13315591	[C/T] T	20 (9.35)	22 (9.65)	1.036	0.548–1.958	0.01	0.913 (P)
**13**	rs874040	[C/G] C	60 (28.3)	70 (29.91)	1.081	0.718–1.628	0.14	0.708 (P)
**14**	rs6859219	[A/C] A	58 (26.61)	57 (23.75)	0.859	0.563–1.311	0.50	0.482 (P)
**15**	rs10499194	C/T—T	77 (37.02)	54 (25.71)	0.589	0.338–0.895	6.21	0.013
**16**	rs3093023	[A/G] A	82 (40.59)	93 (39.41)	0.952	0.649–1.397	0.06	0.800 (P)
**17**	rs394581	[C/T] C	91 (42.52)	105 (49.07)	1.302	0.889–1.906	1.84	0.174 (P)
**18**	rs5029937	[G/T] T	22 (11.96)	25 (10.59)	0.872	0.475–1.603	0.19	0.660 (P)
**19**	rs548234	[C/T] C	52 (25.74)	53 (24.09)	0.915	0.589–1.424	0.15	0.695 (P)
**20**	rs629326	[G/T] G	88 (44.44)	50 (33.33)	0.625	0.402–0.971	4.40	0.0359 (P)
**21**	rs10488631	C/T T/C---C	33 (14.86)	29 (12.83)	0.843	0.493–1.443	0.39	0.533
**22**	rs10739580	[A/C/G/T] T/C	50 (27.17)	52 (33.33)	1.34	0.842–2.133	1.53	0.217
**23**	rs2812378	[C/G/T] A/G---G	53 (24.54)	58 (24.17)	0.980	0.639–1.504	0.01	0.927 (P)
**24**	rs3761847	[A/G] G	90 (45)	70 (40.7)	0.839	0.555–1.267	0.70	0.403 (P)
**25**	rs4750316	[C/G/T] C/G—C	47 (22.38)	46 (19.01)	0.814	0.516–1.285	0.78	0.376 (P)
**26**	rs706778	[A/G] C/T---T	95 (45.67)	107 (49.08)	1.147	0.784–1.678	0.50	0.481 (P)
**27**	rs2104286	A/G T/C—C	38 (17.75)	43 (18.85)	1.077	0.664–1.744	0.09	0.765
**28**	rs540386	[C/G/T] C/T---T	56 (25.93)	69 (29.24)	1.180	0.780–1.786	0.62	0.432 (P)
**29**	rs10683701	[-/ACTT] DEL	81 (37.5)	72 (31.58)	0.936	0.636–1.377	0.11	0.737 (P)
**30**	rs1678542	[C/G] G	67 (31.6)	74 (31.36)	0.989	0.663–1.474	0.00	0.955
**31**	rs763361	[A/C/T] A/T	4 (4.26)	5 (4.72)	1.114	0.290–4.276	0.02	1.000 (F)
**32**	rs4810485	[A/G/T] G/T---T	51 (25)	39 (19.7)	0.987	0.628–1.551	0	0.953 (P)
**33**	rs3218253	[C/T] G>A	61 (28.77)	68 (29.06)	1.014	0.673–1.528	0.00	0.947 (P)

**Table 5:** Genotype and allele frequencies of the SNPs showing an association with RA in the Saudi population, in the RA patients compared to the controls.

SNPs rs1188934	**Variations**	**Control No. (%)**	**Case No. (%)**	**OR**	**CI**	**χ^2^**	***p*** **-Value**
	AA	35 (33.02)	60 (52.6)	2.857	1.248–6.541	6.42	0.011
	GG	20 (18.9)	12 (10.5)	0.035	0.153–0.801	6.42	0.011
	AG	51 (48.11)	42 (36.8)	0.480	0.268–0.861	6.13	0.013
	Allele Frequency						
	A	121 (57.08)	162 (71.05)	1.846	1.244–2.740	9.35	0.002
	G	91 (42.92)	66 (28.95)	0.542	0.365–0.804	9.35	0.002
	Variations	Control No. (%)	Case No. (%)	OR	CI	χ^2^	*p*-value
rs10919563	AA	22 (22.68)	8 (7.62)	0.309	0.128–0.748	7.24	0.007
	GG	57 (58.76)	67 (63.81)	3.232	1.337–7.816	7.24	0.007
	AG	18 (18.56)	30 (28.57)	1.418	0.716–2.807	1.01	0.315
	Allele Frequency						
	A	62 (31.96)	46 (21.9)	0.597	0.383–0.932	5.2	0.023
	G	132 (68.04)	164 (78.1)	1.675	1.073–2.613	5.2	0.023
rs3087243	Variations	Control No. (%)	Case No. (%)	OR	CI	χ^2^	*p*-value
	AA	34 (32.08)	23 (19.83)	0.282	0.126–0.628	9.93	0.002
	GG	15 (14.15)	36 (31.03)	3.548	1.591–7.910	9.93	0.002
	GA	57 (53.77)	57 (49.14)	1.478	0.776–2.815	1.42	0.233
	Allele Frequency						
	A	125 (58.96)	103 (44.40)	0.556	0.381–0.810	9.41	*p* = 0.002
	G	87 (41.04)	129 (55.6)	1.799	1.235–2.623	9.41	*p* = 0.002
rs1980422	Variations	Control No. (%)	Case No. (%)	OR	CI	χ^2^	*p*-value
	CC*	12 (13.4)	22 (18.48)	1.956	0.868–4.407	2.66	0.103
	TT	48 (52.17)	45 (37.81)	0.511	0.227–1.152	2.66	0.103
	TC	32 (34.78)	52 (43.69)	1.733	0.952–3.157	3.26	0.071
	Allele Frequency						
	T	130 (70.65)	96 (40.33)	0.647	0.431–0.972	4.41	0.036
	C	56 (30.43)	142 (59.66)	1.545	1.029–2.322	4.41	0.036
	Variations	Control No. (%)	Case No. (%)	OR	CI	χ^2^	*p*-value
rs10499194	TT	22 (21.15)	10 (9.52)	0.365	0.158–0.843	5.81	0.016
	CC	49 (47.12)	61 (58.1)	2.739	1.186–6.323	5.81	0.016
	TC	33 (31.73)	34 (32.38)	0.828	0.450–1.522	0.37	0.542
	Allele Frequency						
	T	77 (37.02)	54 (25.71)	0.589	0.338–0.895	6.21	0.013
	C	131 (62.98)	156 (74.29)	1.698	1.118–2.58)	6.21	0.013
	Variations	Control No. (%)	Case No. (%)	OR	CI	χ^2^	*p*-value
rs629326	GG	21 (21.21)	21 (28)	0.696	0.327–1.479	0.89	0.345
	TT	32 (32.32)	46 (61.33)	1.438	0.676–3.057	0.89	0.345
	GT	46 (46.46)	8 (10.67)	0.121	0.050–0.290	25.74	<0.0000
	Allele Frequency						
	G	88 (44.44)	50 (33.33)	0.625	0.402–0.971	4.40	0.0359
	T	110 (55.56)	100 (66.67)	1.600	1.030–2.485	4.40	0.0359
	Variations	Control No. (%)	Case No. (%)	OR	CI	χ^2^	*p*-value
rs6859219	AA	9 (8.26)	15 (12.5)	1.282	0.525–3.129	0.30	0.585
	CC	60 (55.05)	78 (65)	0.780	0.320–1.904	0.30	0.585
	CA	40 (36.7)	27 (22.5)	0.519	0.287–0.939	4.75	0.029
	Allele Frequency						
	A	58 (26.61)	57 (23.75)	0.859	0.563–1.311	0.50	0.482 (P)
	C	160 (73.39)	183 (76.25)	1.164	0.763–1.776	0.50	0.482 (P)
rs11586238	Variations	Control No. (%)	Case No. (%)	OR	CI	χ^2^	*p*-value
	CC	66 (60.6)	83 (71.6)	0.838	0.284–2.48	0.1	0.749
	GG	6 (5.5)	9 (7.8)	1.193	0.404–3.521	0.1	0.749
	CG	37 (33.9)	24 (20.9)	0.516	0.281–0.946	4.64	0.031
	Allele Frequency						
	C	169 (77.25)	190 (81.9)	1.312	0.827–2.08	1.33	0.248
	G	49 (22.48)	42 (18.1)	0.762	0.481–1.209	1.33	0.248

## Data Availability

The study materials and datasets are available from the corresponding author upon reasonable request.
